# Molecular characterisation of methicillin-resistant *Staphylococcus aureus* from chronically colonised cystic fibrosis paediatric patients in Brazil

**DOI:** 10.1017/S0950268820001156

**Published:** 2020-05-26

**Authors:** D. C. S. Rodrigues, D. F. Lima, R. W. F. Cohen, E. A. Marques, R. S. Leão

**Affiliations:** 1 Departamento de Microbiologia, Imunologia e Parasitologia, Faculdade de Ciências Médicas, Universidade do Estado do Rio de Janeiro, Boulevard 28 de Setembro, 87, fundos, 3° andar – Vila Isabel, Rio de Janeiro, 20551-030, Brazil; 2 Departamento de Pneumologia Pediátrica, Instituto Nacional da Saúde da Mulher da Criança e do Adolescente Fernandes Figueira, Fundação Oswaldo Cruz, Av. Rui Barbosa, 716 – Flamengo, Rio de Janeiro 20021-140, Brazil

**Keywords:** MRSA, Cystic fibrosis, PFGE, SCC*mec*, *spa* typing, *Staphylococcus aureus*

## Abstract

Persistent methicillin-resistant *Staphylococcus aureus* (MRSA) infection in cystic fibrosis (CF) patients has been associated with a more rapid decline in lung function, increased hospitalisation and mortality. The aim of this study was to evaluate the clonal relationships among 116 MRSA isolates from 12 chronically colonised CF pediatric patients over a 6-year period in a Rio de Janeiro CF specialist centre. Isolates were characterised by antimicrobial resistance, SCC*mec* type, presence of Panton-Valentine Leukocidin (PVL) genes and grouped according to DNA macrorestriction profile by pulsed-field gel electrophoresis (PFGE) and *spa* gene type. High resistance rates were detected for erythromycin (78%) and ciprofloxacin (50%) and SCC*mec* IV was the most common type (72.4%). Only 8.6% of isolates were PVL positive. High genetic diversity was evident by PFGE (39 pulsotypes) and of nine that were identified *spa* types, t002 (53.1%) and t539 (14.8%) were the most prevalent. We conclude that the observed homogeneity of *spa* types within patients over the study period demonstrates the persistence of such strain lineages throughout the course of chronic lung infection.

## Introduction

Cystic fibrosis (CF) is an autosomal recessive disease predominantly in Caucasian populations. Although affecting multiple systems, the chronic obstructive pulmonary disease is the main contributor to morbidity and mortality in such patients [1].

*Staphylococcus aureus* is one of the earliest and most prevalent pathogens isolated from the airways of CF patients. Colonisation with methicillin-resistant *S. aureus* (MRSA) has risen dramatically over the past decade [2] and has been associated with poor clinical outcomes in these patients [3]. According to the Brazilian Cystic Fibrosis Registry 2017, MRSA was the seventh most common microorganism isolated from respiratory cultures of CF patients [4]. A cohort study conducted in USA showed that chronic lung colonisation by MRSA resulted in clinical deterioration with an accelerated decline in lung function compared with patients colonised by methicillin-susceptible strains [5]. In addition, the presence of MRSA was associated with a twofold higher risk of death than for patients without this pathogen [6].

Methicillin resistance is determined by the *mecA* gene, located on the Staphylococcal Cassette Chromosome *mec* (SCC*mec*) element. Currently, 13 SCC*mec* types have been described and types I–V are the most frequently reported, including in CF patients [7, 8]. Characterisation of SCC*mec* type in combination with Multilocus Sequence Typing forms the basis of the international nomenclature of MRSA clones [9, 10]. Several such clones have been identified in CF patients around the world [9, 11, 12].

In addition to MLST, other strain typing techniques have been utilised for molecular epidemiological studies of MRSA, with pulsed-field gel electrophoresis (PFGE) profiling and Staphylococcal Protein A (*spa*) gene typing being among the most widely used [10, 13, 14]. The choice of methods depends largely on the aim and nature of the investigation taking into accounts its advantages and limitations [15]. Generally, a combination of methods is preferred to optimise reproducibility and discriminatory power in order to differentiate epidemiologically related strains from non-related strains.

Numerous virulence-associated factors have been described in *S. aureus,* but notably, expression of Panton-Valentine Leukocidin (PVL), encoded by *lukS* and *lukF* genes, has been associated with the development of invasive lung infections of CF patients due to MRSA strains [9, 16]. Some epidemic lineages of MRSA, are most commonly linked with SCC*mec* IV and often with PVL production, including isolates recovered from patients with CF [9, 12].

There have been few longitudinal molecular epidemiological studies of chronic colonisation by MRSA in CF patients in Brazil. An understanding of the distribution and stability of MRSA clones in these patients is essential for the use of effective treatments and to inform strategies to control their dissemination in order to improve patient survival. This study reports the clonal relationships of MRSA isolates from chronically colonised CF paediatric patients in a specialist centre over a 6-years period.

## Materials and methods

### Patients and samples

This study was carried out on MRSA isolates recovered from respiratory samples from 12 chronically colonised paediatric outpatients attending a CF reference centre in Rio de Janeiro, over a period of 6 years. Patients were classified as chronically colonised according to the criterion of at least three MRSA positive isolates in a 12-month period [5]. All other patients were excluded. In total, 116 isolates were available for study. The number of isolates varied from 4 to 20 per patient and had been recovered from sputum, oropharynx swab and tracheal aspirate samples. The mean age of the patients was 7.3 years (range 2–18), with the mean age at CF diagnosis of 38.6 months.

The study was approved by the institutional board of CEP/CONEP system (CAAE: 36885614.5.0000.5259)

### Identification and antimicrobial susceptibility

Isolates were identified as *S. aureus* by Gram stain, catalase production, mannitol fermentation, coagulase and DNase production, and resistance to cefoxitin. Antimicrobial susceptibility tests were performed by disc diffusion assay and interpreted according to the Clinical Laboratory Standards Institute [17] for the following antimicrobials: gentamicin, chloramphenicol, ciprofloxacin, clindamycin, erythromycin, linezolid, rifampin, tetracycline and trimethoprim/sulfamethoxazole (Oxoid Ltd, Basingstoke, UK). Multidrug resistance (MDR) was defined as resistance to three or more classes of non-*β*-lactam antimicrobials.

### Genotypic characterisation

Polymerase chain reaction (PCR) amplification of genes encoding PVL (*lukS/F*-PV) and SCC*mec* types were determined as described previously [8, 18]. Isolates were genotyped using PFGE following *SmaI* (Fermentas, ThermoScientific, Massachusetts, USA) digestion of chromosomal DNA, according to a published protocol [19]. The computer-assisted analysis was performed using BioNumerics v.7.6 software (Applied Maths, Sint-Martins-Latem, Belgium) and banding patterns were compared by the Unweighted Pair Group Method using the Arithmetic Mean (UPGMA) and the Dice similarity coefficient of 1%. A pulsotype cluster was defined as a group of isolates sharing ≥80% of pattern similarity and assigned primarily as letters A-Z and, subsequently as AA-MM.

Representative isolates (*n* = 81) of the same profile (pulsotypes, presence/absence of genes *lukS*/*F*-PV, SCC*mec* types and antimicrobial resistance patterns) were selected for *spa* gene typing as previously described [20]. The sequencing reactions were performed with the Big Dye Terminator v.3.1 Cycle Sequencing kit (Applied Biosystems, Foster City, USA) and sequenced in an ABI 3500 Genetic Analyzer (Applied Biosystems). Assembled *spa* sequences were analysed using the *spa*-plugin in the Bionumerics v.7.6 software (Applied Maths).

## Results

### Antimicrobial susceptibility

The isolates showed intermediate or full resistance to erythromycin (78.4%), ciprofloxacin (50%), clindamycin (33.6%), rifampicin (25%), chloramphenicol (19%), gentamicin (19%), tetracycline (12.9%) and trimethoprim/sulfamethoxazole (12%). None was resistant to linezolid and 37.1% were classified as MDR.

### Genotypic characterisation

Among the 116 isolates, 18 (15.5%) harboured SCC*mec* II, 14 (12.1%) were SCC*mec* III and 84 (72.4%) were SCC*mec* IV. Ten (8.6%) isolates from four patients (2, 4, 7 and 9) were positive for PVL genes and all were of SCC*mec* IV.

PFGE analysis revealed 39 distinct patterns among the 116 isolates, with the following type patterns being the most frequent: A and FF (*n* = 11), EE (*n* = 9), X (*n* = 8) and CC (*n* = 7). Table 1 shows that the majority of the patients harboured isolates of different pulsotypes over the study period, with the exception of patient 11 whose isolates were consistent of type X. Nine types were shared among the patients, with Y and FF being the most frequent.

**Table 1.** Temporal distribution of the spa types and pulsotypes PFGE among 116 MRSA isolated from cystic fibrosis paediatric patients

Patient	Year 1	Year 2	Year 3	Year 4	Year 5	Year 6
	Isolation date – *spa* typing – pulsotype related					
1		(*n* = 6) 19/02 – t539 – P; 19/02 – t539 – O; 19/03 – t539 – N; 24/06 – t539 – N; 20/08 – Ø – N; 19/11 – Ø – O	(*n* = 3) 15/07 – t539 – P; 21/10 – Ø – N; 16/12 – t002 – HH	(*n* = 5) 24/03 – t539 – N; 18/04 – Ø – T; 20/06 – t539 – T; 18/07 – t539 – II; 19/09 – t539 – Q	(*n* = 2) 16/04 – t539 – Q; 18/08 – t539 – JJ	
2	(*n* = 2) 10/07 – t002 – AA; 30/10 – t002 – U	(*n* = 1) 02/04 – t002–U	(*n* = 2) 01/04 – t002 – EE; 06/05 – t 18 066 – G	(*n* = 1) 05/05 – t18 066 – E	(*n* = 1) 02/02 – t18 066 – E	
3				(*n* = 2) 17/08 – t021 – I; 22/09 – Ø – I	(*n* = 2) 02/02 – t021 – I; 26/04 – t021 – K	
4	(*n* = 2) 03/06 – t1605 – H; 14/08 – t1605 – H	(*n* = 7) 22/01 – t1605 – G; 19/02 – t1605 – H; 19/03 – Ø – G; 16/04 – Ø – G; 16/07 – t1605 – KK; 20/08 – t1605 – F; 15/10 – Ø – F		(*n* = 1) 28/04 – Ø – F		
5		(*n* = 1) 01/10 – t002 – FF	(*n* = 4) 07/01 – t002 – Z; 01/04 – t002 – GG; 28/04 – t002 – GG; 05/05 – t002 – Y	(*n* = 7) 16/03 – t779 – A 19/05 – Ø – A; 22/06 – Ø – A; 07/07 – Ø – A; 18/08 – t779 – A; 24/08 – t002 – L; 06/10 – t779 – C	(*n* = 6) 26/04 – t779 – A; 26/04 – t779 – A; 16/05 – Ø – A; 02/08 – Ø – A; 29/08 – t779 – D; 17/10 – t779 – B	(*n* = 2) 21/02 – Ø – A; 04/04 – Ø – A
6			(*n* = 1) 09/09 – t002 – U	(*n* = 2) 07/07 – t002 – EE; 06/10 – t8784 – R	(*n* = 2) 26/04 – t002 – FF; 04/10 – t002 – FF	(*n* = 2) 10/01 – t8784 – R; 20/02 – t002 – FF
7	(*n* = 1) 25/06 – t002 – S	(*n* = 3) 22/05 – Ø – CC; 16/07 – Ø – CC; 20/08 – Ø – CC	(*n* = 1) 05/04 – t002 – CC	(*n* = 3) 21/02 – t002 – CC; 07/07 – t1154 – GG; 06/10 – t1154 – GG	(*n* = 2) 2/07 – t1154 – Y; 01/1 – Ø – Y	(*n* = 1) 10/01 – t1154 – GG
8	(*n* = 5) 10/04 – t002 – BB; 15/05 – Ø – BB; 31/07 – t002 – Y; 28/08 – t002 – HH; 12/12 – Ø – HH	(*n* = 3) 12/01 – t002 – HH; 16/02 – t002 – LL; 13/04 – t002 – MM	(*n* = 1) 15/12 – t539 – M			
9	(*n* = 1) 20/08 – t002 – DD	(*n* = 2) 08/01 – t1154 – U 02/04 – t002 – CC	(*n* = 1) 07/01 – t002 – CC		(*n* = 1) 02/02 – t318 – J	
10	(*n* = 2) 30/07 – t002 – Y; 10/12 – Ø – FF	(*n* = 4) 12/02 – t002 – FF; 30/03 – Ø – FF; 25/06 – t002 – O; 20/08 – Ø – FF				
11			(*n* = 3) 16/06 – t002 – X; 11/08 – t002 – X; 16/12 – Ø – X	(*n* = 3) 19/05 – Ø – X; 21/07 – t002 – X; 15/09 – Ø – X	(*n* = 1) 01/11 – Ø – X	(*n* = 1) 28/02 – Ø – X
12			(*n* = 3) 10/05 – t002 – EE; 26/05 – t002 – EE; 20/09 – Ø – V	(*n* = 6) 21/02 – t002 – EE; 16/03 – t002 – W; 28/04 – t002 – FF; 19/05 – t002 – W; 21/07 – Ø – FF; 15/09 – Ø – FF	(*n* = 2) 01/08 – Ø – EE; 26/11 – Ø – EE	(*n* = 2) 20/02 – Ø – EE; 01/04 – t002 – EE

**Fig. 1.**
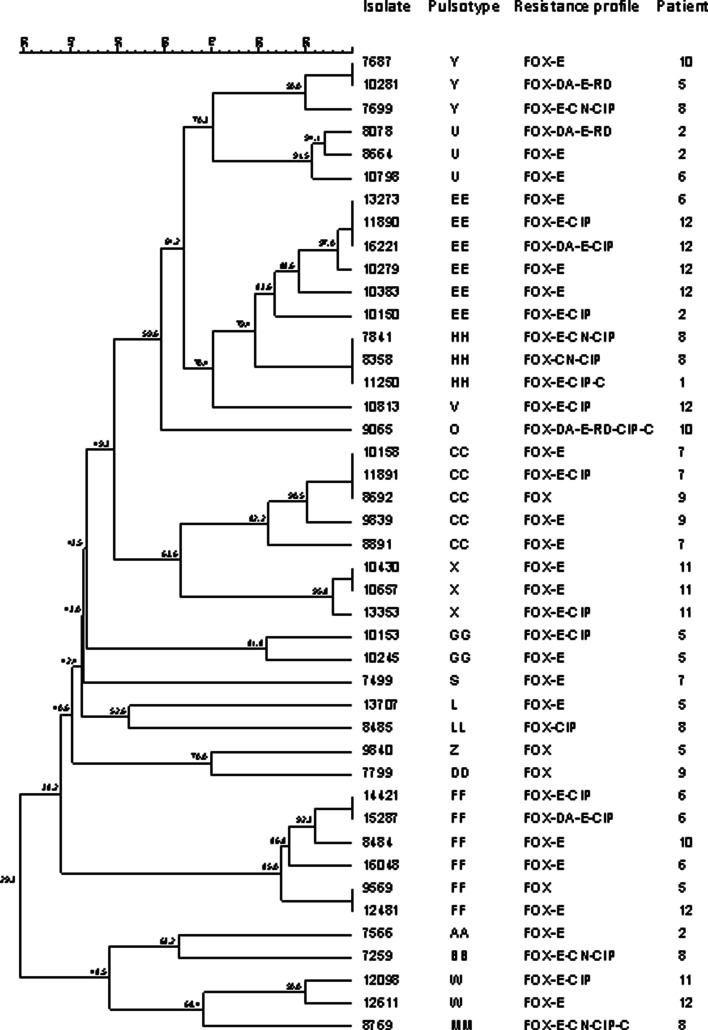
Analysis of SmaI macrorestrincton profiles of 43 MRSA spa type t002 isolates from cystic fibrosis paediatric patients.

Nine *spa types* were identified among the 81 selected isolates; the most prevalent being t002 (*n* = 43, 53.1%) and t539 (*n* = 12, 14.8%). A novel *spa* type was identified (t18 066, *n* = 3), from patient 2. Overall, there was a predominance of a specific *spa* type for each patient and patients 3, 4, 10, 11 and 12 harboured isolates of the same *spa* type over different years. Interestingly, patients 2 and 7 were initially colonised by strains of *spa* t002 but in later years showed the emergence of types t 18 066 and t1154, respectively, which replaced the initial strain (Table 1). As *spa* t002 was more widely distributed among different patients, it is notable that these isolates proved highly diverse and were discriminated into 19 pulsotype patterns (Fig. 1).

Six *spa* types were associated with SCC*mec* type IV, which accounted for 74.1% (*n* = 60) of isolates. However, SCC*mec* II was identified only in types t539 and t8784, and SCC*mec* III in t779. No *spa* type was associated with more than one type of SCC*mec*. All isolates of t002 harboured SCC*mec* IV and most (*n* = 33, 76.7%) were non-MDR. In contrast, all t539 isolates were SCC*mec* II and also MDR. PVL genes were detected in four *spa* types: t002 (1/43), t318 (1/1), t1605 (3/5) and t 18 066 (2/3) (Table 2).

**Table 2.** Molecular typing and antimicrobial resistance profile among 81 MRSA isolated from cystic fibrosis patients

Patient	Isolates (*n*)	*lukS/F* (*n*)	SCC*mec* (*n*)	*spa* type (*n*)	Antimicrobial resistance profile (*n*)
1	12	Negative (12)	II	t539 (11)	FOX-DA-E-CN-RD-CIP-TE-C (1)^a^
					FOX-DA-E-RD-CIP-C (5)^a^
					FOX-DA-E-RD-CIP (4)^a^
					FOX-DA-E-CIP-C (1)^a^
			IV	t002 (1)	FOX-E-CIP-C (1)
2	7	Negative (4)	IV	t002 (4)	FOX-DA-E-RD (1)^a^
					FOX-E-CIP (1)
					FOX-E (2)
		Positive (2) Negative (1)		t18 066 (3)	FOX (3)
3	3	Negative (3)	IV	t021(3)	FOX-DA (1)
					FOX (2)
4	5	Positive (3) Negative (2)	IV	t1605 (5)	FOX-CIP (2)
					FOX (3)
5	13	Negative (13)	IV	t002 (6)	FOX-DA-E-RD (1)^a^
					FOX-E-CIP (1)
					FOX-E (2)
					FOX (2)
			III	t779 (7)	FOX-DA-E-CN-RD-CIP-TE-SXT-C (2)^a^
					FOX-DA-E-CN-RD-CIP-TE-SXT (3)^a^
					FOX-DA-E-CN-CIP-TE-SXT-C (1)^a^
					FOX-DA-E-CN-CIP-TE-SXT (1)^a^
6	7	Negative (7)	IV	t002 (5)	FOX-DA-E-CIP (1)^a^
					FOX-E-CIP (1)
					FOX-E (3)
			II	t8784 (2)	FOX-DA-E-CIP-C (2)^a^
7	8	Positive (1) Negative (3)	IV	t002 (4)	FOX-E-CIP (1)
					FOX-E (3)
		Negative (4)		t1154 (4)	FOX-E-CIP (1)
					FOX-E-C (1)
					FOX-E (2)
8	7	Negative (7)	IV	t002 (6)	FOX-E-CN-CIP-C (1)^a^
					FOX-E-CN-CIP (3)^a^
					FOX-CN-CIP (1)
					FOX-CIP (1)
			II	t539 (1)	FOX-DA-E-RD-CIP (1)^a^
9	5	Negative (3)	IV	t002 (3)	FOX-E (1)
					FOX (2)
		Positive (1)		t318 (1)	FOX (1)
		Negative (1)		t1154 (1)	FOX-E (1)
10	3	Negative (3)	IV	t002 (3)	FOX-DA-E-RD-CIP-C (1)^a^
					FOX-E (2)
11	3	Negative (3)	IV	t002 (3)	FOX-E-CIP (1)
					FOX-E (2)
12	8	Negative (8)	IV	t002 (8)	FOX-DA-E-CIP (1)
					FOX-E-CIP (3)
					FOX-E (4)

FOX, cefoxitin; C, chloramphenicol; CIP, ciprofloxacin; DA, clindamycin; E, erythromycin; CN, gentamicin; LZD, linezolid; RD, rifampin; SXT, sulfamethoxazole-trimethoprim; TE, tetracycline.

a Multidrug resistance (MDR).

## Discussion

To our knowledge, this is the first study of a molecular characterisation of isolates from CF patients chronically infected by MRSA, in Brazil. According to the American Cystic Fibrosis Registry, between 2000 and 2010, the prevalence of MRSA in CF patients has increased dramatically [2] and in recent years it has been suggested that the possibility of MRSA eradication in CF patients presenting with chronic infection is significantly lower when compared to patients with intermittent colonisation [21]. Epidemiological typing studies of MRSA in chronically infected CF patients are relatively scarce [22, 23] and such data, particularly from longitudinal studies, remain necessary to better understand the patterns of acquisition and dynamics of strain populations over time. Regarding antibiotic susceptibility, the high resistance rates to erythromycin, ciprofloxacin and clindamycin observed in our study were consistent with the literature on MRSA from many different types of infections [24, 25]. Indeed, other studies in paediatric CF patients colonised by MRSA have also shown a high rate of resistance to these agents [22, 23]. Likewise, the great majority (72.4%) of our isolates were SCC*mec* IV and proved to be more susceptible to the non-*β*-lactam antibiotics tested. Other studies in MRSA colonised CF patients have also reported a high prevalence of SCC*mec* IV [23, 26]. This finding was expected as the latter element was also frequent among CF MRSA isolates from a previous study by our group [9]. SCC*mec* IV is characterised as being of relatively small size, a property which facilitates its transfer between strains of *S. aureus*, allowing a higher dissemination ability [27].

A study conducted in the USA with a large number of paediatric CF patients with chronic MRSA infection found that patients colonised by SCC*mec* II strains received more antimicrobials and had a higher probability of developing a chronic infection than those with MRSA-SCC*mec* IV strains [28]. Moreover, a Brazilian study with non-chronic CF patients reported a high prevalence of multiresistant MRSA harbouring SCC*mec* III, which was more common in patients with CF than in non-CF patients [29].

In addition to antimicrobial resistance, virulence factors have a significant negative influence on MRSA infections and the PVL toxin, in particular, poses a threat to the health of the patients, especially when associated with necrotising pneumonia [30]. In this study, only 10 (8.2%) isolates from four of the 12 patients were positive for PVL genes and all of them harboured SCC*mec* IV, which is frequently associated with toxin-producing strains [30]. Although a low frequency of PVL positive isolates has been reported in CF patients [22, 23, 31], such strains are more likely to be associated with lung inflammation as the toxin is considered a potent inducer of inflammation and cytotoxicity [16, 32].

There are relatively few studies in patients with CF infected with MRSA that have documented the permanence of strain lineages over time [11, 23, 26]. The majority of our patients harboured different pulsotypes and this was particularly evident for the predominant *spa* type t002. This diversity might be related to microevolutionary events that take place in the hostile airway environment in CF patients [23]. This resulted in a markedly low association with *spa* types since several of the latter grouped in different pulsotypes. Indeed, isolates of t002 comprised 19 PFGE patterns, some of which differed greatly from each other as illustrated in Fig. 1. Such diversity of DNA macrorestriction patterns within an apparent clonal lineage, as defined by *spa* type, has been previously reported [33], a finding which draws into question the practical value of PFGE for the type identification of CF MRSA. Nevertheless, it is relevant to note that the high diversity shown by PFGE does not exclude the permanence of an overarching lineage among isolates within a patient as different pulsotypes might group in the same, or different, lineage depending on the discriminatory ability and inherent stability of the primary molecular technique that is used [11]. It is well established that point mutations, insertions, deletions, acquisition, or loss of plasmids can cause changes in an enzyme restriction site and thus account for differences in macrorestriction patterns of epidemiologically related strains [15]. Indeed, the population of *S. aureus* is likely to be continuously evolving as a consequence of the lung environment typical of CF, therefore, it is quite conceivable that bacterial populations in this context will consist of closely, as well as distantly, related subpopulations descending from an original infecting strain [34].

In this study, *spa* t002 was the most frequent and widely distributed lineage, being identified in 10 of 12 individuals. Similar results were reported in the USA, where *spa* t002 (36%) was also most prevalent in paediatric CF patients [22] and likewise in Argentina CF patients [23]. Indeed, three of our patients (10, 11 and 12) yielded only type t002 strains which is supportive of continued persistence in these patients. Similarly, *spa* t021 and t1605, both SCC*mec* IV, were restricted to patients 3 and 4, respectively. It is notable that each of the foregoing isolates was of SCC*mec* IV, which is recognised as having a higher dissemination ability [27]. Furthermore, the often-reported association between the presence of SCC*mec* IV and PVL genes [12, 16, 18] as well as wider antimicrobial resistance was not observed.

The substitution of strain *spa* types by another type was evident among four patients (2, 5, 7, 8). Antimicrobial resistance does not appear to be a determinant factor for the replacement of MRSA types among paediatric patients included in this study. Tables 1 and 2 shows the substitution and antimicrobial resistance profile for patient 2 t002→t18 066 (more susceptible); patient 5 t002→t779 (more resistant); patient 7 t002→t1154, (similar); patient 8 t002 →t539 (similar).

Isolation of intermittent types was observed in patients 6 (t002, t8784) and 9 (t002, t1154, t318). In patient 1, clearly chronic for t539, sporadic isolation of t002 was evident in year 3. These types occur separated in sequence, however, there is overlap between successive periods of isolation over the 6-year study (Table 1). This indicates that different strain types coexisted in the lung, some of which were detectable but their presence or absence was possibly not only due to simple replacement, but a likely consequence of sample and cultural variability.

A decrease in resistance to antimicrobials was observed only in isolates from patient 5 which suggests that replacement of MRSA strain types was not necessarily a consequence of acquired resistance due to antimicrobial selective pressures. Other factors such as climate, social, geographical location and other pulmonary microbial flora may also have played a role. Likewise, the intermittent isolation of some *spa* types, as for patients 6 and 9, may reflect possible competition between strains and allow the preferential detection of those in greater number in the sputum sample.

Isolates classified as MDR pose a primary concern for CF patients since they limit the choice of antimicrobial agents for treatment leading to a greater risk of poor clinical outcomes and death [35]. Overall, the resistance profiles of patient's isolates showed a similar distribution but types t539, t779 and t8784 exhibited the highest resistance profiles. The other *spa* types harboured the SCC*mec* IV element and, as expected, most isolates were among the more antimicrobial susceptible group. Isolates of SCC*mec* types I, II and III often harbour additional genes which confer resistance to non-*β*-lactam antimicrobials. However, SCC*mec* IV and V strains generally lack other resistance determinants other than the *mecA* gene and thus are more associated with a higher degree of susceptibility to non-*β*-lactam agents [35].

Our study has a number of limitations, namely the small number of patients from a single CF centre, the variable number of isolates for each patient and the lack of corroboration of the PFGE profiling of isolates with another genomic approach such as multilocus sequence typing or whole-genome sequencing. Nevertheless, this work has helped to improve our understanding of the distribution and dynamics of MRSA strain lineages in chronic colonisation in our patients. These data have informed the design of guidelines for effective strategies to limit and control the dissemination of MRSA in our centre and therefore contribute to better patient survival.

In conclusion, this study examined the predominance of specific *spa* types and sub-populations of MRSA isolates from a cohort of 12 CF patients over a 6-year period. We found few correlations of strain types with antimicrobial resistance patterns and the presence of SCC*mec* types; relatively few isolates harboured PVL genes. *Spa* gene typing confirmed the persistence, with some overlap, of strain lineages over extended periods in some patients, with type t002 occurring in 10 of the patients. The value of DNA macrorestriction profiles for longitudinal studies of MRSA isolates from CF is therefore questionable and should be replaced by sequence-based techniques. Nevertheless, the demonstrated persistence of *spa* gene lineages over a long period emphasises their ability to adapt and survive in the hostile environment of the airways in CF.
